# Rethinking the approach to viability monitoring in seed genebanks

**DOI:** 10.1093/conphys/cox009

**Published:** 2017-03-04

**Authors:** Fiona R. Hay, Katherine J. Whitehouse

**Affiliations:** 1 T.T. Chang Genetic Resources Center, International Rice Research Institute, Los Baños, Philippines

**Keywords:** Genebank, seed conservation, seed longevity, viability monitoring

## Abstract

Seed viability monitoring, usually through a germination test, is a key aspect of genebank management; a low viability result triggers the regeneration of an accession in order to ensure that the genetic diversity of the accession is conserved and available for distribution. However, regular viability monitoring of large collections is costly in terms of seeds, labour and other resources. Genebanks differ in how they conduct their viability monitoring and how they collect, manage and store the data that are generated. In this article, we propose alternatives to the current norm of conducting an initial germination test soon after arrival at the genebank and then testing after regular, set storage intervals, as recommended in the Food and Agriculture Organization's Genebank Standards for Plant Genetic Resources for Food and Agriculture. We use real data from the International Rice Genebank (held at the International Rice Research Institute) to illustrate some of the issues regarding the accuracy and reliability of germination test results, in particular when they are used to predict the longevity of a seed lot in storage and to set viability monitoring intervals. We suggest the use of seed storage experiments on samples of seeds to identify which seed lots from a particular crop season to test first. We also give advice on the use of sequential testing schemes potentially to reduce the number of seeds used for viability testing; the use of tolerance tables to identify unlikely results when samples are subdivided into replicates; and what data to include in a genebank management database to improve the management of seed collections.

## Teaser

A key operation of most seed genebanks is the regular monitoring of the viability of accessions through a germination test. In this article, we suggest ways in which the viability monitoring could be changed to save seeds and resources and reduce error and bias.

## Introduction

A key target in the management of seed collections is to keep the seeds alive. Seeds should be kept in conditions where they remain viable for as long as possible and must be regenerated before they lose the ability to germinate. However, they should not be regenerated unnecessarily, because there is a high cost of regeneration and the risk of loss of genetic integrity through factors such as genetic shift, genetic drift, pollen contamination, seed contamination and mislabelling (Sackville Hamilton and Chorlton, 1997; Kameswara Rao *et al*., 2016). Normally, therefore, the viability of seeds should be monitored to guide timely regeneration. However, with an ever-increasing number of accessions held in the hundreds of genebanks around the world, most of which are supported by public funding, there is pressure to optimize management procedures and introduce efficiency measures that reduce costs.

One area of operations that incurs considerable costs in the management of orthodox seed collections is the viability monitoring. For the vegetable germplasm conserved at The World Vegetable Center, Schreinemachers *et al*. (2014) estimated that, in 2012, the cost of germination/viability testing was USD 104.10 per accession tested. This was based on tests on relatively few accessions (59) and, clearly, there would be economies of scale, as well as variation depending on the accessions (species) being tested. A costing study carried out in 2011 estimated that the average per accession cost of germination testing across the various seed crops stored in the international genebanks of the CGIAR was USD 7.80 (based on 2009 figures and costs; CGIAR Consortium Office, 2011). Aside from the costs of supplies, equipment and labour, it is also costly in terms of requiring seeds that might otherwise be kept in storage or used for distribution. Indeed, a ‘holy grail’ of seed conservation research is to develop a non-destructive method for estimating viability (e.g. Colville *et al*., 2012; Fu *et al*., 2015) which, given the diversity of varieties, crops and wild species in *ex situ* seed conservation, seems a somewhat unachievable goal unless a truly universal marker can be found and exploited. Furthermore, genebank managers perhaps err on the side of caution and might never completely trust any correlative indicator, to avoid the risk of being the custodian who unintentionally fails to maintain the diversity of a collection. It has also been suggested that we need better monitoring of seed ageing to supplement traditional germination tests (Fu *et al*., 2015), the implication of which might be that the extent of deterioriation needs to be assessed before a viability (germination) standard is reached or perhaps that the accuracy of a standard germination test is insufficient.

The genebank standards published by the Food and Agriculture Organization of the United Nations (FAO) describe the procedures to follow to manage collections of plant genetic resources, as well as summarizing some of the science underpinning those procedures (FAO, 2014). The standards cover conventional orthodox seed collections, as well as field, *in vitro* and cryopreserved collections, and they are the standards, perhaps minimal standards, that international genebanks are expected to adopt. There are four standards relating to the viability monitoring of orthodox seeds, two pertaining to the initial testing and two to the monitoring (FAO, 2014). For each of these pairs, one standard is for the timing of seed testing and the other is for the viability percentage. In the case of the latter, the standard for initial viability is 85% ‘for most seeds of cultivated crop species’, although lower values may be adopted for seed lots of some accessions or species, particularly those of wild origin. The viability threshold (standard) for regeneration is also 85%, although again there is a ‘get-out clause’ for difficult accessions. The standards that relate to timing state that the initial test should be conducted within a year of arrival at the genebank and that monitoring test intervals should be one-third of the time predicted for viability to decrease to 85% of initial viability [i.e. through the use of the Ellis and Roberts (1980) seed viability equations] or, if a predication cannot be made, every 5 or 10 years, depending on whether seeds are expected to be short or long lived, respectively.

This article presents some of the problems with the way in which viability monitoring of genebank-stored seeds is currently conducted and how and what data are collected and saved. We suggest options for overcoming these problems, including the use of tolerance tables, sequential testing schemes, image analysis to score germination tests and the use of seed storage experiments to rank seed lots being placed into genebank storage according to relative longevity, and present a flowchart showing when these options might be used. We also make suggestions regarding the collection and storage of viability monitoring data and encourage genebank managers to take time to understand viability monitoring data.

## Problems with current viability monitoring

### Non-optimal germination test procedures

What a genebank manager ideally needs to manage collections better is a test at the time the seeds are placed into storage that either predicts when the seed lots will reach the viability threshold (standard; the minimal viability percentage required) or that ranks the seed lots for relative longevity. This is what the initial germination test is supposed to indicate, at least when dealing with accessions of the same species, which are therefore assumed to lose viability at the same rate when stored in the same conditions (seed moisture content, temperature and gaseous environment). It is important that the initial test does indeed provide an accurate indication of the viability of the seed lot at the time they are placed into storage (if seeds are held for long periods after arrival at the genebank before they are placed into storage, a test may also be needed soon after arrival) and that appropriate measures are taken if the result is below the viability standard in use, i.e. that there is ‘active management’ and decision-taking, rather than mere collection or only cursory consideration of data.

In particular, treatments to overcome dormancy and for germination should be optimized for the species being tested (Davies *et al*., 2015a, b). The International Seed Testing Association (ISTA) publishes ‘rules’ for testing a range of important agricultural and horticultural crop species (ISTA, 2016a), many of which will be priority species for *ex situ* conservation in genebanks. There is also a vast literature on dormancy and germination for species not covered by ISTA (Baskin and Baskin, 2014; Royal Botanic Gardens Kew, 2016). However, dormancy may be released or induced during storage, meaning that dormancy-breaking and/or optimal germination conditions may change (Crawford *et al*., 2007; Pérez-García *et al*., 2009; González-Benito *et al*., 2011).

**Figure 1:**
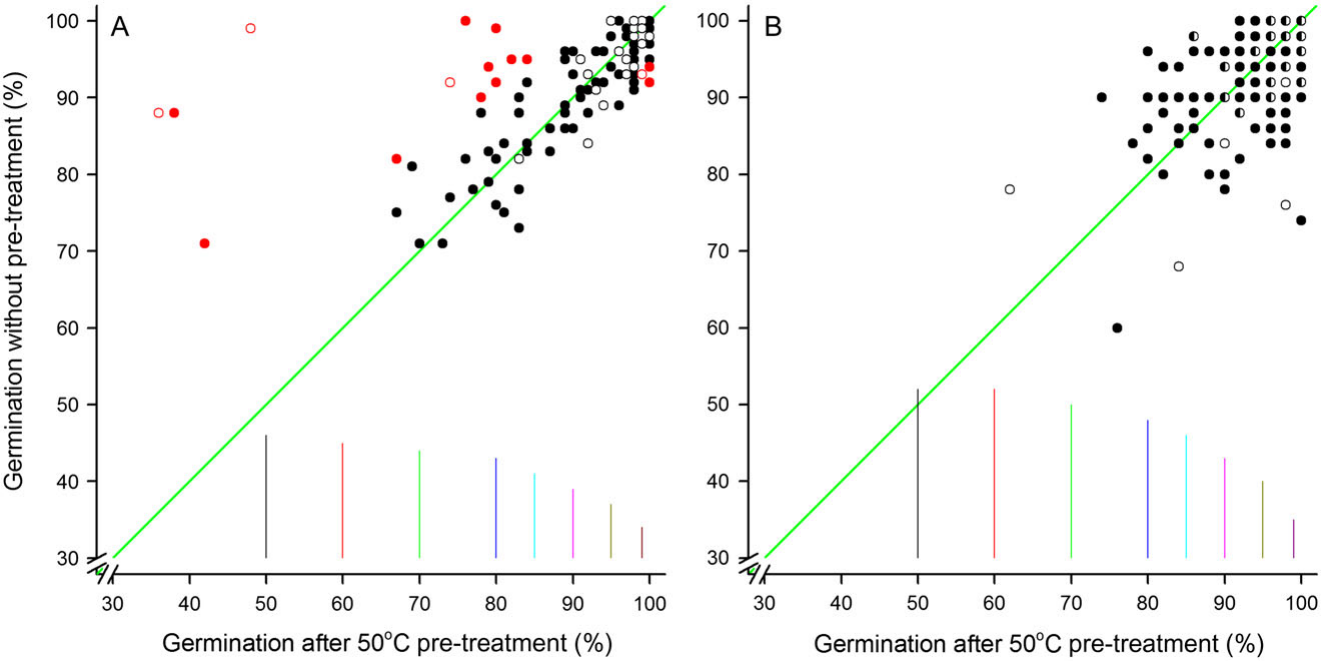
Germination of samples of rice accessions stored in either the active (at 2–4°C; **A**) or base collections (−20°C; **B**) in the International Rice Genebank for between 5–6 (open symbols) and 10–36 years (solid symbols). In B, half-filled symbols indicate results that were observed for both storage period ranges. Seeds from the same sample were germinated either with or without the standard dry-heat treatment (50°C for 7 days). The diagonal green lines show the line of equality, i.e. where there is no difference in the germination depending on whether or not seeds were given the pre-treatment. For illustration, the red symbols in A show those observations where the two results, if the seeds had been treated identically, would have been out of tolerance according to Miles (1963) as available in the Germination Tool Box (ISTA, 2016b) and based on two replicates of 100 seeds each. The vertical lines in A are the maximal tolerated difference (5% probability) between two replicates of 100 seeds each with average germination percentage as indicated by the horizontal axis. The vertical lines in B are the maximal tolerated difference between two replicates of 50 seeds each.

In the case of the *Oryza sativa* accessions held in the International Rice Genebank, the routine viability testing has involved a dry-heat treatment (7 days at 50°C) before equilibration to ambient conditions and sowing for germination at 30°C; this is one of the pre-treatments recommended by ISTA (2016a) for breaking dormancy in rice. However, recent tests, carried out on a random sample of seed lots stored in the medium-term (active; at 2–4°C) and long-term (base; at −20°C) collections, have shown that this treatment may be detrimental to the germination of the seeds, particularly for those stored in the active collection (Fig. 1A). For seeds stored in the base collection, the pre-treatment in general did not have a detrimental effect nor did it increase germination (Fig. 1B). In the case of *O. sativa*, any dormancy that is present in the seeds at harvest may be lost during drying (Hay and Timple, 2016) or during storage, through ‘after-ripening’, and in future, for the *O. sativa* accessions at the International Rice Genebank, a dry-heat pre-treatment will not be used for older seed lots. Again, this emphasizes the need for genebank managers to take time to examine germination viability monitoring data critically.

If germination is low, a cut-test can also be used to identify whether dormancy is likely to be an issue (Davies *et al*., 2015a), or a viability test *sensu stricto*, for example using tetrazolium chloride solution (ISTA, 2016a), may be performed. If it is confirmed that the initial viability of a seed lot is indeed too low, the most appropriate management decision is likely to be to reject the seed lot, unless it will be difficult to acquire a new seed lot to represent the accession/species.

### Sampling and scoring bias

Whether or not a viability test is also conducted, the germination test result will be only an estimate of the seed lot viability based on a sample of seeds from the seed lot, and the reliability of the estimate will largely depend on the sample size, i.e. the number of seeds that are used for the viability test. The number of seeds sampled for a viability monitoring test will depend on the number of seeds in the bulk sample and how easy it is to replenish stocks. Davies *et al*. (2015a) suggest that for accessions with very few seeds, a sample size as small as 10 seeds could be used; the sample size increases to 25 or 25–50 if there are more seeds. For species for which it is difficult to collect large numbers of seeds, viability testing may be dispensed with altogether (Davies *et al*., 2015a). FAO (2014) recommends, based on ISTA testing protocols, that 200 seeds should be used for the initial germination test, at least, but further suggests that 50 seeds may be a more realistic sample size for viability monitoring owing to limited resources and/or seeds.

**Figure 2:**
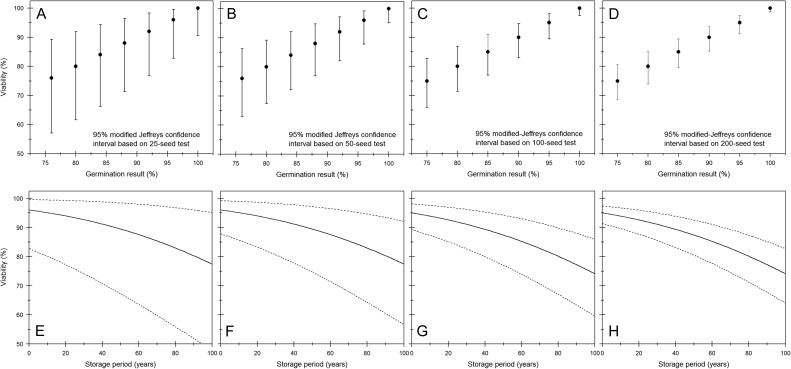
Confidence intervals give an indication of the reliability of a germination test result (proportion or percentage germination) as an estimate of the whole seed lot viability. (**A–D**) Ninety-five per cent modified Jeffrey's confidence intervals [Brown *et al*., 2001; calculated using EpiTools (AusVet Animal Health Services, 2016)] for sample sizes of 25, 50, 100 and 200 seeds and germination results ≥75%. (**E–H**) The predicted survival curves for rice seeds stored at 7% moisture content and 3°C with initial viability of 96 (E and F) or 95% (G and H) or the lower and upper limits of the 95% confidence intervals of these proportions for the corresponding sample size (as indicated in the upper graphs).

The confidence interval can be used to indicate a level of confidence in the estimated viability and varies with the sample size and depending on the result (proportion of germinated seeds; Fig. 2). For example, if 25 seeds are sown and 23 (92%) germinate, the lower and upper 95% modified-Jeffrey's confidence limits (Brown *et al*., 2001) would be 98.3 and 76.7%, respectively (Fig. 2A). If the seed lot viability was at these limits, there would be very different survival curves (Fig. 2E), Indeed, the probability that the seed lot viability is already ≤85% is 0.254 (based on an exact binomial test in GenStat release 18.1; VSN International Ltd, Hemel Hempstead, UK). Using more seeds for an initial test would increase the reliability of the result; hence, it might provide better prediction of the expected progress of viability loss in subsequent storage (Fig. 2).

Ideally, good seed sampling procedures will be followed when taking a sample for germination testing, to avoid a biased sample. For example, settling in a seed container may occur if there is variation in individual seed size and/or density and, depending on the species, seed size may influence germinability (e.g. Cideciyan and Malloch, 1982; Winn, 1985; Aiken and Springer, 1995; Vera, 1997). Further discussion on why a sample should be taken at random and advice on how to do so is provided by Ellis *et al*. (1985).

There is some evidence that technical staff responsible for scoring germination tests, which is often done in a systematic way, examining each replicate in turn (and probably in a predefined order) before moving on to the next seed lot, are unconsciously biased in ensuring that replicate scores are similar (Deplewski *et al*., 2016). These conclusions were drawn from data from mainly ISTA-accredited laboratories. Where genebank viability monitoring tests are carried out by genebank staff, it is not beyond the realms of possibility that as well as unintentionally minimizing the difference between replicates, there may be a predisposition to avoid germination results that are close to or below the viability standard.

### Reliance on the viability equations for predictions

The genebank standards recommend using the Ellis and Roberts (1980) seed viability equations to predict the time for viability to decrease to 85% of initial viability and set monitoring intervals at one-third of this time (FAO, 2014). As well as the inconsistency in using 85% of initial viability rather than an absolute value of 85% and the unreliability of the initial germination test as a measure of true viability (see above), there is the assumption in this recommendation that the parameters of the seed viability equations, if determined (they have only been determined for a handful of species), do not vary among seed lots within a species, or in cases such as rice, variety group (Ellis *et al*., 1992). However, it has been known for some time that there can be variation in the rate of viability loss between seed lots of the same species (or in the case of rice, variety group), for example owing to the maturity (or range in maturity) of the seeds at harvest (Hay and Probert, 1995; Hay *et al*., 2015a), and indeed, apparently independent of the maturity of the seeds at harvest (Whitehouse *et al*., 2015; Whitehouse, 2016). This makes it impossible to set appropriate seed lot-specific viability monitoring schedules based on the initial germination result and rate of viability loss predicted using the Ellis and Roberts (1980) viability equations.

**Figure 3:**
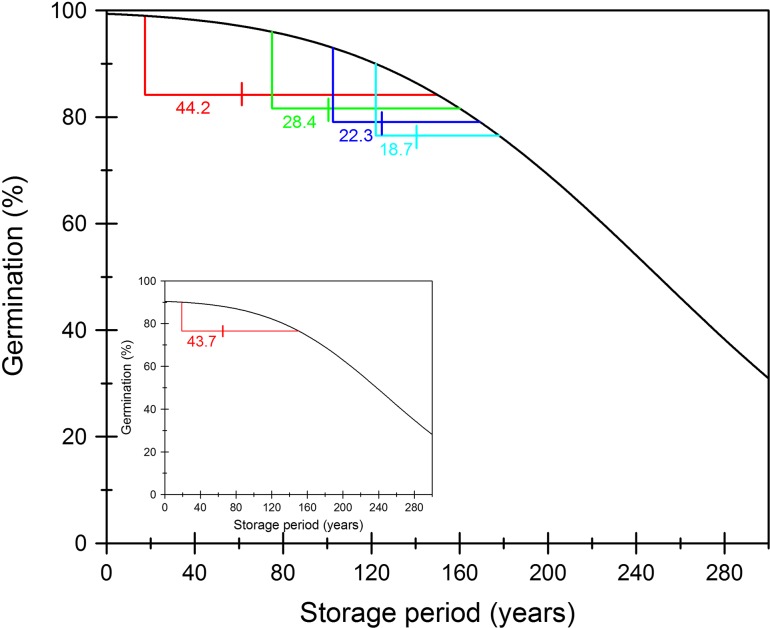
Setting viability test intervals at one-third of the time it takes for viability to decrease to 85% of the initial viability means that sampling intervals will vary. Predicted survival curves are shown for rice seeds stored at 7% moisture content and 3°C (estimated using the Seed Information Database; Royal Botanic Gardens Kew, 2016), with varying initial viability, as follows: 99% (red), 96% (green), 93% (blue) and 90% (cyan). Theoretical sampling intervals (red, green, blue and cyan numbers, respectively) are shown for each survival curve, calculated as one-third of the time predicted for viability to decrease to one-third of the initial viability (in years). The inset graph shows an example where it may be more appropriate to use 85% of initial viability for determining sampling intervals, when there is a proportion (in this case, 10%) of non-respondents in the population of ageing seeds. Hence, although the initial viability (as a proportion of seeds sown) is close to 90%, the sampling interval is almost double that of the seed lot in the main graph with 90% initial viability.

Furthermore, setting viability intervals at one-third of the time it takes for viability to decrease to 85% of the initial viability would mean that intervals would vary both between seed lots within a species and between species (Fig. 3). Although this could, of course, be programmed into genebank management software, a more simple exercise for single-crop genebanks, it could lead to very variable monitoring intervals, with perhaps a risk of missing some seed lots that should be tested, depending on how genebank managers identify which seed lots are due for testing. Furthermore, as discussed elsewhere (Hay *et al*., 2015a), a standard of 85% of initial viability may genuinely be needed only if it is expected that there is a proportion of the population of seeds that cannot germinate and which was never part of the ageing population of seeds (‘non-responders’; Fig. 3, inset).

## Solutions

### Tolerance tables

The seed sample drawn for testing is usually subdivided for sowing as two or more ‘replicates’, although in fact, it would be preferable to draw the seeds for the replicates independently. When more than one replicate sample of seeds is sown for the viability monitoring test, there is likely to be some variation in the number of seeds that germinate in each replicate. In cases with high variation, in particular when three or more replicates are used, it may be tempting simply to discard the odd result. Rather, tolerance tables could be used to identify whether the whole test should be repeated.

Tolerance tables show the maximal range in the number of germinated seeds in each replicate that can be accepted based on the number of replicates, number of seeds in each replicate and overall (or mean) germination percentage (Miles, 1963; Ellis *et al*., 1985; ISTA, 2016b). The tolerated range also depends on the desired probability, i.e. the proportion of tests where the observed results could be obtained through random variation. The tolerated range is greatest when the overall germination is 50% and decreases as the mean germination percentage decreases or increases to 0 or 100%, respectively (Fig. 1).

As well as informing decisions on individual monitoring results, tolerance tests could also be looked at as a whole. If a high proportion of tests are failing the tolerance test, it suggests that there is some other factor causing dispersion in the results. For example, if there is a pronounced temperature gradient in the test environment and replicates are not rotated, one replicate may be at suboptimal germination conditions for the duration of the test.

### Sequential testing schemes

Sequential testing schemes are a useful way to save staff time and seeds (Ellis *et al*., 1985; Kameswara Rao *et al*., 2006). Reducing the number of seeds used for viability monitoring is particularly important when the quantity of seeds available is limited. A smaller sample of seeds is sown at first and then, if germination is too low, depending on the viability standard and how many seeds are sown, another sample is taken and then the cumulative results are evaluated. In theory, fewer seeds are likely to be used overall than if a fixed sample size (of more seeds) was used from the outset. This is particularly the case if viability is still expected to be high. Ellis *et al*. (1985) recommend using a larger sample if viability is already expected to be approaching the viability standard. One drawback of a sequential testing procedure is that accessions may be repeatedly, in close succession, withdrawn from storage for sampling for germination testing. It may also be more difficult to document, and the staff resources involved in retrieving and sampling accessions may mean that in the end, there is not much labour saving.

### Overcoming bias

When scoring viability monitoring tests, it may be useful for evaluators to see previous results, to spot trends or odd results. However, to avoid any subconscious bias, it may be better to make scorers blind to the historical data, at least until the new data have been entered, as well as randomizing the scoring of seed lots and accessions. Unconscious bias could also be avoided by out-sourcing germination testing, a practice followed by the Centre for Genetic Resources, The Netherlands (CGN), although this still does not guarantee reliability of results. Van Hintum and van Treuren (2012) reported that when a random selection of accessions were sampled twice for independent germination testing by ISTA-certified agencies, 18.1% of the paired tests (not the replicates within a test) showed greater dispersion than expected.

Alternatively, especially for single- or few-crop genebanks, using machine vision/image analysis to score germination results may remove any potential human bias. The use of image analysis by seed testing authorities to follow the progress of seed germination has increased in recent years (Joosen *et al*., 2010; Wagner *et al*., 2011; Matthews *et al*., 2012), although the aim is often to evaluate seed vigour (speed and uniformity of germination, hence likely field emergence), rather than the final proportion of germinated seeds. Nonetheless, automation of the scoring of seed germination could save some staff time and reduce scoring bias or inconsistencies in genebanks and also provide information on the speed of germination, which might be useful to genebank users, in particular breeders wanting to develop varieties with greater seed and seedling vigour.

### Revision of monitoring intervals

The Genebank Standards recommend that ‘*As soon as a significant decline is detected, monitoring intervals should be reduced to ‘fine tune’ the prediction of time to reach the viability standard*’ (FAO, 2014). This suggests that there might be more and more tests as the viability standard is approached, to ensure that the viability standard value is not missed. At its extreme, it could be interpreted as needing to reset the viability monitoring interval to one-third of the new prediction of time taken for viability to reach 85% every time a germination test result is received. Owing to the expected error in the germination results, it might even mean that viability test intervals for a single seed lot lengthen as well as shorten. Hence, interpreted literally, it is likely to waste time and resources, in particular seeds, unless it is expected that there will be a sudden and catastrophic loss of viability as the seed lot approaches the viability standard (or indeed, if the seed lot started with low germination). In reality, most seed survival curves do not have a sharp ‘shoulder’; there is normally variation between individual seeds in the time during storage at which they lose the ability to germinate (e.g. Priestley *et al*., 1985; Sinniah *et al*., 1998; Crawford *et al*., 2011); indeed, this is true even when deliberate attempts are made to reduce the inherent variation within a seed lot (Hay *et al*., 2010).

**Figure 4:**
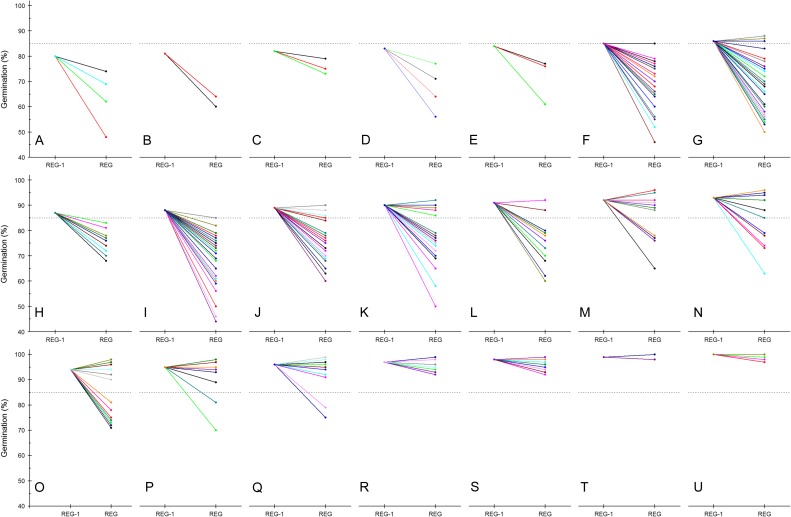
Germination data for rice accessions held in the medium-term (active) collection of the International Rice Genebank and regenerated in the 2015 dry season. The germination data are the last germination test result (REG) and the result of the test preceding that last test (REG-1) for the seed lots being replaced by regeneration in the 2015 dry season. Hence, most of the REG tests were conducted in 2014 and the REG-1 tests in 2008–09. The seed lots tested were from crop years in 1979–2011. The data are sorted according to the result of the REG-1, ranging from 80% (**A**) to 100% (**U**). Some of the data are hidden because of repeat results. For example, in **H**, data are shown for 12 accessions (seed lots) with 87% germination and REG-1 and 68–83% germination at REG. The horizontal dashed lines correspond to 85% germination.

This suggested adjustment also leads to the question of how much leeway there should be around the set viability standard with respect to deciding to regenerate. In practice, most genebanks are probably making a decision to regenerate based on a range of viability monitoring test results (and some seed lots are, of course, replaced because of dwindling stocks and may still have viability greater than the genebank standard; Fig. 4). This range is likely to vary depending on the typical rate of viability loss for the species being stored, the conditions of storage and the existing monitoring schedule. The range may also be a consequence of the error in the preceding result when viability appeared to be above the standard.

**Figure 5:**
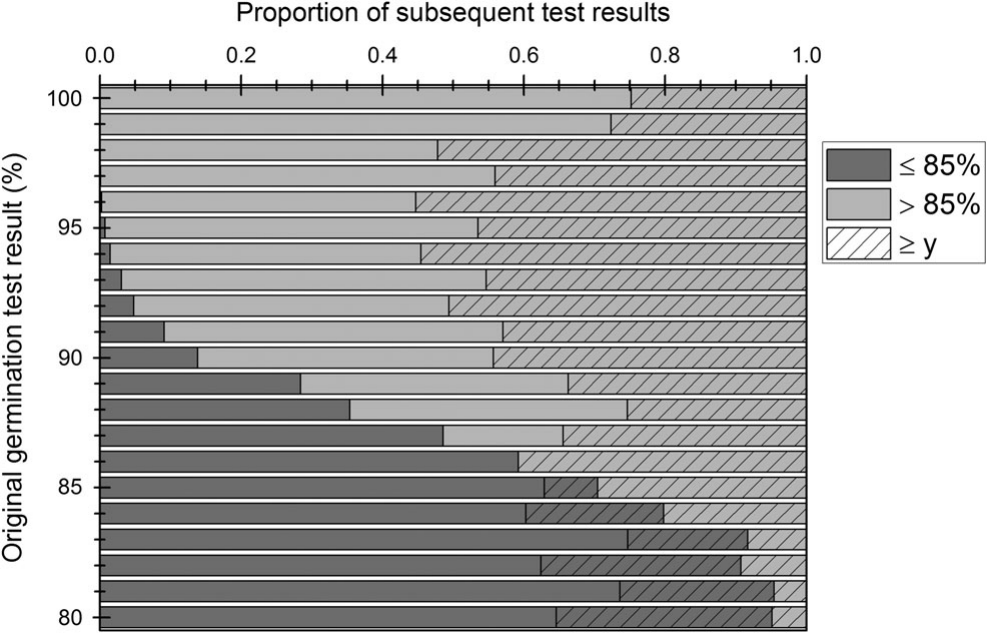
The proportion of subsequent viability monitoring test results, carried out 4–6 years after the ‘original’ germination test, showing ≤85% germination or ≥86% germination, sorted according to the original germination test result. Also shown is the proportion of results that are greater than or equal to the original germination test result (≥y). The data are for *Oryza sativa* accessions stored in the active collection of the International Rice Genebank. The original germination test results are the results of tests conducted between 1 January 2000 and 31 December 2009 and include both initial and monitoring test results. Before October 2012, regeneration was carried out when viability reached 85% of the initial germination; therefore, some seed lots with <85% germination in the original germination test were maintained in storage and tested subsequently.

For example, considering the germination data for rice accessions held in the International Rice Genebank that were tested in 2014 and subsequently regenerated in the 2015 dry season, seed lots showing 88% germination in the test before 2014 (conducted in 2008–2009) had a germination result in 2014 ranging between 44 and 85% (Fig. 4I). The predicted time for viability to decrease from 88 to 85% for *indica* rice seeds stored at 7% moisture content and 3°C is 14 years; for *japonica* rice seeds, the predicted time is 6 years (predictions estimated using the seed viability equations module of the Seed Information Database; Royal Botanic Gardens Kew, 2016). Hence, a result as low as 44% within 5–6 years is unexpected. In fact, the data in Fig. 4 suggest that there can appear to be a sudden and sharp decline in viability, even when the preceding test result is as high as 90–93% (Fig. 4K–N). Of course, these data represent only a small subset of all the tests that were conducted in 2008–2009 and 2014. Considering all the data for seed lots tested in 2000–2009, the proportion of seed lots with ≤85% germination in the subsequent test does increase the lower the original result (i.e. result observed in 2000–2009), but a considerable proportion (41%) of the seed lots with, e.g. 86% germination, showed ≥86% germination in the subsequent test (Fig. 5). For seed lots with 87% germination in the original test, 49% showed ≤85% germination in the subsequent test, 16% showed 86% germination and 34% showed ≥87% germination. These data suggest that a shortening of test intervals may be advisable, perhaps only when the test results are 86 or 87%. Of course, there will always be other constraints influencing whether additional tests are carried out (availability of resources to conduct more germination tests), or indeed, whether seed lots are regenerated as soon as they reach the viability standard (availability of resources for regeneration).

### Seed storage experiments

In order to gain a better understanding of the storage potential of a seed lot when the seeds are being placed into genebank storage, rather than simply taking a sample of seeds for an initial viability test, a larger sample could be taken and used for a seed storage experiment. Seed storage experiments involve raising the moisture content of the seeds, sealing them inside moisture-proof packets (usually laminated aluminium foil packets of the type that is often used for storage in the genebank) and placing them at a relatively high temperature. Individual packets (typically a total of 8–12 packets are prepared for each seed lot) are sampled at regular intervals to test the seeds for germination. The comparative longevity protocol used by the Millennium Seed Bank Partnership (Newton *et al*., 2014) was designed to screen seed lots of diverse species for relative longevity in a controlled ageing environment (in equilibrium with 60% relative humidity and 45°C). The germination data from such experiments are traditionally modelled using probit analysis (Hay *et al*., 2014), thereby fitting the first viability equation (Ellis and Roberts, 1980), as follows:

$$v=K_{\text{i}}-p/\text{σ},$$

where *v* is the viability in probits (normal equivalent deviates, NED) after *p* days storage, σ is the time for viability to decrease by one probit, and *K*_i_ is the estimated initial viability in probits, which can be transformed to a percentage value. The confidence interval for the estimate of *K*_i_ (i.e. of initial viability), because a generalized linear model is fitted, is potentially smaller than when a simple initial viability test is carried out. The confidence interval will vary depending on how many sampling times there are during the storage experiment and how many seeds are tested at each sampling time, and indeed, on the initial viability and the rate of loss of viability.

**Figure 6:**
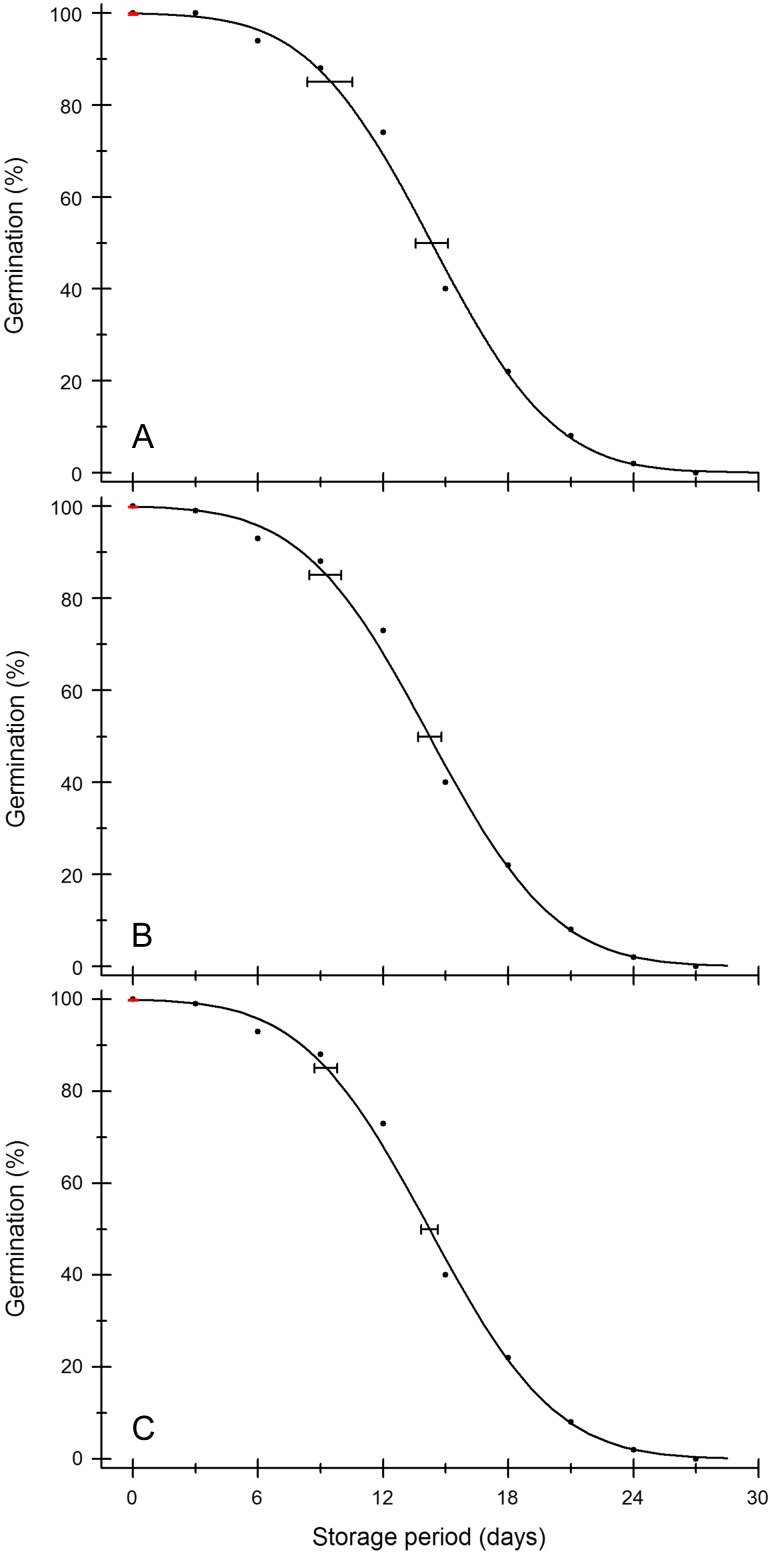
Survival curves based on the analysis of a storage experiment on rice seeds of accession IRGC117279 harvested at 38 days after flowering (Hay *et al*., 2015a). Seeds were stored at 10.9% moisture content and 45°C and sampled for a germination test every 3 days. (**B**) the original data, with samples of 100 seeds, fitted by probit analysis. The *x*-error bars are the lower and upper 95% confidence limits for the time for viability to decrease to 85 or 50% (*p*_85_ and *p*_50_, respectively). The y-error bars (red lines) at time 0 days are the 95% confidence limits for *K*_i_ (probit initial viability converted to percentage values). (**A** and **C**) The same data adjusted to samples of 50 or 200 seeds, respectively, with corresponding fitted survival curves and confidence limits. For the 50-seed data set, the data for three observations were rounded up because it would not be possible for half a seed to germinate.

For example, on analysis of the data from a storage experiment carried out on seeds of rice accession IRGC117269 harvested at 38 days after flowering from Hay *et al*. (2015a), in which there were six observations (using 100 seeds each), with germination ranging from 95% down to 0% over 15 days, the estimate for *K*_i_ was 1.960 probits (97.5%), with lower and upper limits of 1.669 probits (95.2%) and 2.251 probits (98.8%), which compares with a Jeffrey's 95% binomial confidence interval of 94.6–99.0 if 195 seeds germinated in a 200-seed test (97.5%) or of 96.0–98.5 if 585 seeds germinated in a 600-seed test (likewise 97.5%). Likewise, re-analysis of storage experiment data for accession IRGC117279 from the same paper (Hay *et al*., 2015a), with 100 seeds sown at each sampling time and with germination ranging from 100% at the start of storage down to 0% after 27 days, resulted in an estimate for *K*_i_ of 2.984 probits, equivalent to 99.858%, and lower and upper confidence limits of 2.647 and 3.321 probits, 99.594 and 99.955%, respectively (Fig. 6), which would compare with Jeffrey's 95% binomial lower and upper confidence limits of 99.534 and 99.989% if 999 of 1000 seeds germinated for an initial germination test, or 99.069 and 99.978% if 499 of 500 seeds germinated. Indeed, the advantage of doing a storage experiment to estimate the initial viability (i.e. *K*_i_), rather than simply a high-seed-number initial germination test, is more apparent when viability is very high (99–100%). As these values indicate, high estimates of *K*_i_, in every practical sense, do not reflect higher viability but a longer lag period before viability starts to decline.

Of course the advantage of conducting a seed storage experiment is that it is not only *K*_i_ that is estimated. The results can be used to rank seed lots according to the time for viability to decrease to 50% (*p*_50_; Probert *et al*., 2009; Merritt *et al*., 2014) or, perhaps more relevant to genebanks, to 85% (*p*_85_), or indeed, any other percentile. The relative longevity ranking of seed lots based on data from a seed storage experiment is expected to be similar to that for seed lots in long-term storage, i.e. seed lots that are identified as losing viability quickly in the storage experiment will also lose viability quickly during conservation storage (Probert *et al*., 2009). A modification of the standard longevity protocol, using fewer seeds, has also been developed to screen for short-lived seed lots only (Davies *et al*., 2016). The estimates of *p*_50_ were reliable even when the number of samples taken, over the same total storage period, was reduced from 10 (of 50 seeds each) to four (two of 25 and two of 50 seeds). Likewise, re-analysis of data for rice from Hay *et al*. (2015a) show that while the standard errors of the estimates for *p*_85_ and *p*_50_ increase when the number of seeds used for each sample time is reduced (Fig. 6) and if the number of data points is reduced (to ≤4), the estimates are in reasonable agreement (Supplementary Fig. 1). Correlations between *p*_85_ and *p*_50_ estimates when all the collected data were included in the analysis compared with including only four or fewer observations are significant (Spearman's rank correlation coefficient = 0.96, *P* < 0.001, excluding pairs where estimates were missing for the ≤4-samples test). Hence, the results of an optimized ‘streamlined’ storage experiment (Davies *et al*., 2016) could be used to identify which seed lots, when placed into genebank storage, should be tested first.

Even if it is not possible to obtain estimates of *p*_85_, for example, if seed lots reach low viability very quickly, or conversely, if there is little loss in viability over the experimental storage period, some ranking for longevity would be possible. Only when seed lots identified as being the shortest lived in the storage experiment start to reach the viability threshold (standard) during genebank storage would the next-shortest-lived set of seed lots start to be tested. If seeds of the same seed lot are stored in both medium-term and long-term collections, the shortest-lived seeds in the medium-term collection, which is usually at a higher temperature than the long-term collection, would be monitored first and when the viability standard was reached, as well as initiating the testing of the next-shortest-lived seed lots in the active collection, testing of the long-term-stored seeds of the shortest-lived seed lots would also commence. This approach could be adopted both within and between species. Adopting such a system would require some investment of labour to start conducting seed storage experiments and collecting *p*_85_ data for all the seed lots going into storage from a particular crop season. Having an automated germination scoring system in place might facilitate running initial storage experiments. The use of storage experiments would also depend on the extent of variation in longevity of the cohort as to whether it could save resources in the long term. The data shown in Supplementary Fig. 1 are for rice seed lots that differ in terms of seed maturity at harvest; hence, the magnitude of the variation in *p*_85_ is perhaps inflated, although again, data from Whitehouse (2016) suggest that even within the species and taking into account maturity, seed lots would differ in relative seed longevity to an extent that would mean that testing in batches sorted according to relative longevity is practicable.

## Collecting and using viability monitoring data

For viability monitoring data to be useful, it is important that all the data are entered and retained in the database, ideally the same database that has all the other accession-related data, including passport and characterization data, which may be helpful to identify why particular groups of accessions are behaving in a certain way during storage. Although, from a management point of view, it is perhaps only the current (most recent) germination result that is needed for making decisions (e.g. acceptable viability for distribution, need for regeneration), without any historical data it is impossible to know what is happening during storage. In particular, it would not be possible to identify trends with respect to changes (increase or decrease), or indeed, lack of change, in germinability. If replicates are used for a viability monitoring test, the result of each replicate should be recorded, along with the number of seeds sown, replicate by replicate, in case there is miscounting at the time of preparing the germination test or empty seeds are identifiable only at the end of the test. Knowing how many seeds are used in each viability test is important for any statistical analyses, for example between pairs of results or to compare a result against a reference value (e.g. the viability standard), or for probit analysis if the Ellis and Roberts (1980) viability equations are being fitted to the data. It is also important if a sequential testing scheme is followed or if tolerance limits are used. Sequential testing schemes have been calculated and published only for certain set values, for example, 20, 25, 40, 50 or 100 seeds (Ellis *et al*., 1985). If the formulae for sequential schemes and tolerance testing could be incorporated into the database, then genebank managers could be informed immediately that further testing is necessary.

If the species being tested has seed dormancy and different dormancy-breaking treatments and germination conditions are tried for subsamples of seeds taken at the same viability monitoring interval (the subsamples may be taken over a period of weeks or even months as different tests are tried), the results of all the tests should be entered into the database, together with all the test conditions (Bone *et al*., 2003), not least for stock management reasons and to understand the fate of seeds taken out of storage. In such situations, it may be necessary to include a field that indicates which combination of test conditions produced results that can be considered reliable indicators of viability (i.e. with no dormancy in the non-germinated seeds), as is the case for the seed bank management database used by the Millennium Seed Bank (J. B. Dickie, personal communication).

Viability monitoring data, as well as being an essential aspect of quality control for genebanks, do potentially serve another function; providing information on the relative longevity of seeds in genebank storage (Walters *et al*., 2005; Crawford *et al*., 2007; Hay *et al*., 2013, 2015b, c; Agacka *et al*., 2014). However, one drawback of using genebank viability monitoring data to understand relative seed longevity is that often the data (i.e. germination percentage) span only a relatively small part (e.g. 85–100%) of the response variable (0–100%); hence, probit analysis, which is used to fit the Ellis and Roberts (1980) viability equations, is of limited value. One alternative is to look at the proportions of seed lots with germination less than or equal to a critical value, such as 85%, for different groups of seed lots, perhaps based on accession origin, regeneration location or crop year (Hay *et al*., 2015b). This can give a useful indication of overall performance of groups of seed lots, as well as highlighting problem accessions that have low germination, perhaps independent of storage period. Nonetheless, there is clearly a need to explore or even develop other statistical procedures that can be used to make statistically valid predictions based on narrow data sets and/or revise monitoring intervals as data are captured.

**Figure 7:**
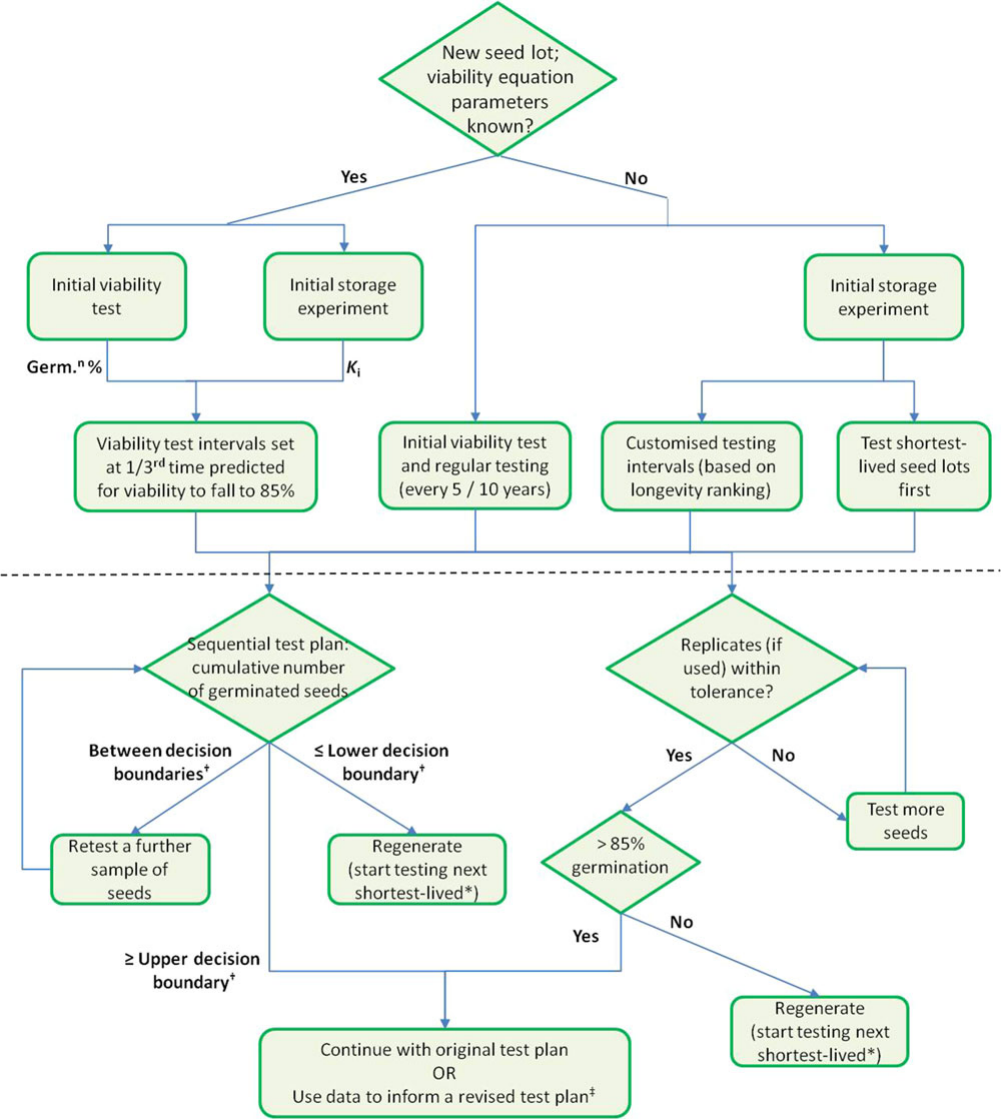
Flow chart showing how a viability monitoring schedule might be set based on an initial viability test or on an initial storage experiment on a sample of the seeds for each seed lot being prepared for genebank storage. If the parameters of the Ellis and Roberts (1980) viability equation are known, intervals could be set based on predicted longevity and either the initial germination (‘Germ.^n^ %’) or the estimate for *K*_i_, the theoretical initial viability in probits, from an initial storage experiment. Alternatively, the results of an initial storage experiment could be used to rank seed lots for relative seed longevity and monitoring plans set accordingly. The bottom half of the chart (below the dashed line) shows the possible decision process once a seed lot has been tested, depending on whether a single sample is tested and a sequential test plan followed or whether replicates are sown and tolerance tables used. ^†^Refers to the decision boundaries of a sequential test plan (see Ellis *et al*., 1985). *If seed lots are tested in batches based on relative longevity ranking from seed storage experiments, once the shortest-lived seed lots start to reach the regeneration standard, testing of the next cohort of seed lots based on the longevity ranking should commence. ^‡^Further research is required to develop algorithms that effectively adjust monitoring intervals to avoid unnecessary usage of seeds.

Another problem with using genebank data to gain an understanding of seed longevity is that many genebanks have been in operation and following defined protocols for a relatively short period of time (perhaps 50 years at most), given the longevity of seeds or many species, particularly crop species, in genebank storage. Furthermore, over the decades, it is almost inevitable that changes have been made to storage conditions, handling procedures and, of course, germination testing regimes (see e.g. Walters *et al*., 2005; Hay *et al*., 2013). Nonetheless, the fact that many genebanks, or more importantly, many seed lots are now a few decades old means that the rates of regeneration because of declining viability are likely to be starting to increase, and there is a more pressing need to make improvements and efficiencies with respect to viability monitoring in order to redirect resources to regeneration and post-harvest handling, including, perhaps, the routine running of storage experiments on a sample of seeds as each seed lot is prepared for genebank storage. Nonetheless, consideration of reliable genebank data, where available, in the context of our current extent of understanding of seed longevity in genebank storage, means that genebank managers should be starting to devise—and customize—more sophisticated monitoring schedules. At the same time, it is clear that there is a need to generate more data on seed longevity and how it can vary within and between species. Indeed, as a highly plastic trait (Leprince *et al*., 2016), it is important that we increase our understanding of how to control various factors that might negatively impact longevity. This might be a by-product of incorporating storage experiments into routine genebank management (Fig. 7). The flowchart shown in Fig. 7 suggests some options for using the data from an initial standard storage experiment or from a viability test, combined with existing tools [the Ellis and Roberts (1980) viability equations, sequential testing schemes and tolerance testing], to set an initial monitoring schedule. The nature of any revision of a test plan, as monitoring data are collected, is a focus of future work. Indeed, it is at this point where evidence from other measures of the extent of seed ageing may be incorporated, on a species-by-species basis, to optimize the timing of further sampling or even to decide when to regenerate without conducting another germination test.

## Supplementary material

Supplementary material are available at *Conservation Physiology* online.

## Supplementary Material

Supplementary Data
