# Supplementary figures and images for: Rethinking the approach to viability monitoring in seed genebanks
Source: Conserv Physiol. 2017 Mar 4;5(1):cox009. doi: 10.1093/conphys/cox009 (PMC5356937; doi:10.1093/conphys/cox009)

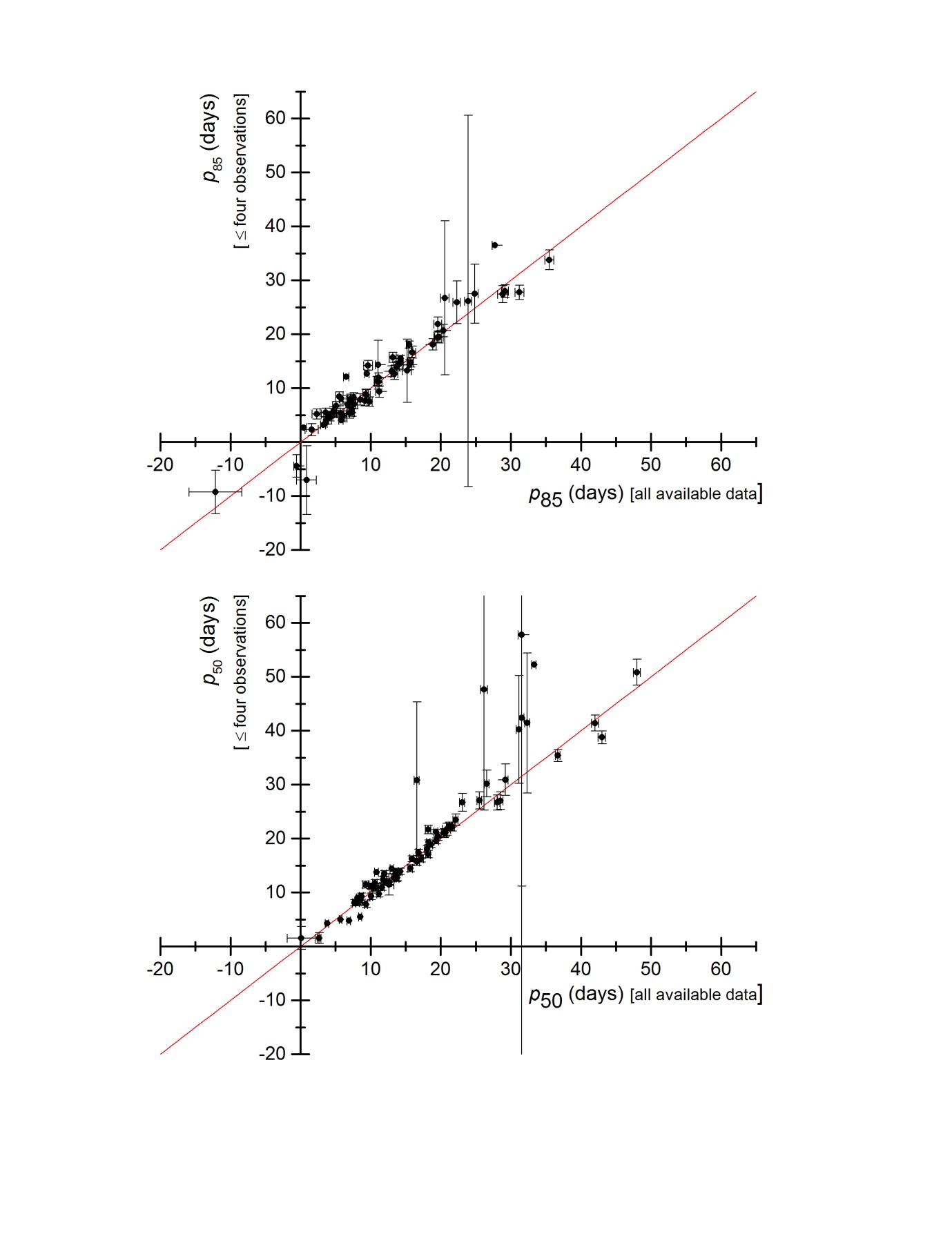

Supplement: Supplementary Data [file CONPHYS-2016-079R1HayandWhitehouseSupplementaryfigure.docx]
